# A Breakthrough in Photocatalytic Wastewater Treatment: The Incredible Potential of g-C_3_N_4_/Titanate Perovskite-Based Nanocomposites

**DOI:** 10.3390/nano13152173

**Published:** 2023-07-26

**Authors:** Rashmiranjan Patra, Pranjyan Dash, Pradeep Kumar Panda, Po-Chih Yang

**Affiliations:** 1 Department of Chemical Engineering and Materials Science, Yuan Ze University, Taoyuan 32003, Taiwan; rashmitheone05@gmail.com; 2 Department of Chemical Engineering and Biotechnology, National Taipei University of Technology (Taipei Tech), Taipei 10608, Taiwan; pranjyandash@gmail.com

**Keywords:** g-C_3_N_4_, titanate-based perovskites, heterojunction, photocatalysis, wastewater treatment, photodegradation

## Abstract

Water pollution has emerged as a major global environmental crisis due to the massive contamination of water resources by the textile dyeing industry, organic waste, and agricultural residue. Since water is fundamental to life, this grave disregard puts lives at risk, making the protection of water resources a serious issue today. Recent research has shown great interest in improving the photocatalytic performance of graphitic carbon nitride (g-C_3_N_4_) for wastewater treatment. However, the photocatalytic removal activity of pure g-C_3_N_4_ is poor, owing to its minimal surface area, fast recombination of photo-generated electron–hole pairs, and poor light absorption. Recently, titanate perovskites (TNPs) have attracted significant attention in both environmental remediation and energy conversion due to their exceptional structural, optical, physiochemical, electrical, and thermal properties. Accordingly, TNPs can initiate a variety of surface catalytic reactions and are regarded as an emerging category of photocatalysts for sustainability and energy-related industries when exposed to illumination. Therefore, in this review article, we critically discuss the recent developments of extensively developed g-C_3_N_4_/TNPs that demonstrate photocatalytic applications for wastewater treatment. The different synthetic approaches and the chemical composition of g-C_3_N_4_/TNP composites are presented. Additionally, this review highlights the global research trends related to these materials. Furthermore, this review provides insight into the various photocatalytic mechanisms, including their potential impact and significance. Also, the challenges faced by such materials and their future scope are discussed.

## 1. Introduction

The dramatic urbanization and industrialization that have occurred over the past few decades have profoundly altered not only the way humans live but also the way other species on this planet coexist. Although this has transformed the technology at our fingertips, which benefits human civilization in numerous ways, it also has a significant negative impact on nature and natural assets, including human and animal health. Throughout the world, every living thing depends on water, which is an invaluable asset. Nearly 70% of the earth’s surface is covered in water, but only 3% of this water is classified as clean. Even though this fact is widely acknowledged in human society, numerous industries continue to pollute water to meet rising human needs. Water is utilized in a wide variety of applications, not only in households, but also in the industrial and agricultural sectors. Approximately two million tons of untreated water were produced by industrial and agricultural discharges in 2003, according to the United Nations World Water Assessment Program (UN WWAP) [1]. The textile and pharmaceutical industries are the two biggest contributors to worldwide water pollution. The textile industry reportedly produces 700,000 tons of dyes worldwide each year, representing more than 10,000 different types of dyes [2,3]. Because they are stable in the presence of light, heat, and other environmental variables, the vast majority of these colored organic dyes are released into the water and persist there for long periods [4]. Aquatic ecosystems are additionally subjected to long-lasting, colorless, stable organic contaminants (emerging pollutants) from the pharmaceutical industry. Aquatic and terrestrial ecosystems face the highest risks from the discharge of these organic pollutants, dyes, and pharmaceutical pollutants in wastewater.

Numerous methods, including adsorption [5], membrane filtration, and advanced oxidation processes [6], are being developed by scientists and researchers to address this issue. However, none of these can eliminate these pollutants from water. Moreover, these methods are limited to a lab scale due to their high cost. These wastewater treatment methods must be cost-effective and environmentally friendly to be used on a large scale. In this context, photocatalysis might be an effective way to solve these problems. Due to its effectiveness in removing pollutants as well as its cost and environmental friendliness, semiconductor photocatalysis is a widely used method for wastewater treatment [7]. It is well-known for degrading a wide range of toxic pollutants from wastewater, including dyes, pesticides [8,9,10,11], antibiotics, heavy ions [12,13,14,15], and other organic pollutants [16,17,18].

Among the various semiconductors, a non-metallic, visible-light-activated semiconductor known as graphitic carbon nitride (g-C_3_N_4_) has received significant attention as a potential material for use in a variety of environmental remediation applications. The structural components of g-C_3_N_4_ include s-triazine units, which are connected by tertiary amines to create a conjugation system. Along with having a tunable energy bandgap of approximately 2.7 eV [19], it also possesses excellent electronic, optical, and thermal properties in different aqueous media [20]. Due to these unique characteristics, g-C_3_N_4_ has been extensively investigated as the most fascinating visible-light-activated photocatalyst in numerous studies [21,22,23]. Nevertheless, their practical use is constrained by a high rate of charge carrier recombination, ineffective solar energy harvesting, a lack of active sites for interfacial reactions, an insufficient specific surface area, and poor charge mobility [24]. ABO_3_ titanate perovskites (TNPs), on the other hand, such as BaTiO_3_, SrTiO_3_, CaTiO_3_, BiTiO_3_, and others, have excellent photocatalytic properties, such as a narrow bandgap [25,26], high charge transfer mobility [27], and the ability to use ferroelectricity, which is beneficial for charge separation. The g-C_3_N_4_ and these titanate perovskite materials can be combined into a single heterostructure, which can overcome each of their individual performance limitations and improve photocatalytic wastewater treatment.

The main objective of this review paper is to investigate the application of these nanocomposites in wastewater treatment. Here, we will cover the various synthesis methods that can be used for manufacturing these nanocomposites. As we try to comprehend the photocatalytic cleaning mechanism and explore various parameters that affect their performance, we will also discuss current research and advancements in this area of study. In order to make better use of these nanocomposites, this paper will discuss their potential future scope and any obstacles that must be overcome.

## 2. Photocatalytic Wastewater Treatment

### 2.1. Basic Principles of Photocatalysis

**Figure 1:**
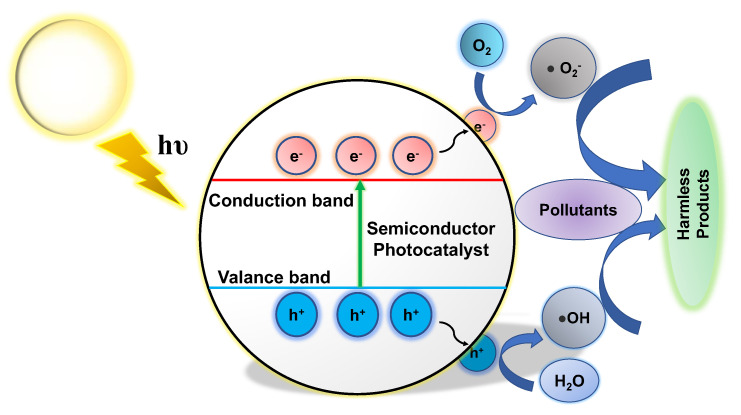
Overview of the photocatalytic mechanism in a semiconductor photocatalyst.

In photocatalysis, when semiconducting materials are exposed to light, chemical reactions are initiated. Photocatalytic wastewater treatment uses light to treat wastewater [28]. In this process, the photocatalyst absorbs photons with energies higher than its bandgap, facilitating the formation of electron–hole pairs (Figure 1). These charge carriers subsequently undergo numerous interactions with adsorbed species on the catalyst surface or in the surrounding environment, resulting in the breakdown and mineralization of contaminants [29]. The photocatalytic process involves three major steps: light absorption, charge separation, and reactive species production. When light is absorbed, electrons in the valence band move to the conduction band and leave behind positively charged holes [30]. The photogenerated electrons and holes can go to the catalyst’s surface, where redox reactions occur with adsorbed species, such as water and organic contaminants. As a result, reactive species are created, such as hydroxyl radicals (•OH) and superoxide radicals (•O_2_^−^), which have powerful oxidizing or reducing properties that can help to break down the pollutants [7,31,32,33,34].

### 2.2. Advantages of Photocatalytic Wastewater Treatment

Compared to conventional treatment techniques such as adsorption [5], membrane filtration, and advanced oxidation processes [6], photocatalytic wastewater treatment has several benefits, such as (a) enhanced degradation: photocatalysis degrades a variety of organic contaminants, including resistant and hazardous substances, through advanced oxidation processes. Compared to conventional treatment approaches, this results in better removal efficiency [35]. (b) Versatile catalysts: depending on the specific target pollutants and environmental factors, a variety of semiconductor materials can be employed as photocatalysts [36]. (c) Utilization of solar energy: photocatalysis uses solar energy as the driving force for pollutant degradation, providing a sustainable and renewable approach to wastewater treatment [37]. (d) Minimal chemical necessities: in contrast to chemical-based processes, photocatalysis only needs a photocatalyst and light source, which minimizes the need for additional chemicals and lowers operating costs. (e) Potential for resource recovery: the photocatalytic process can make it easier to convert pollutants into safe byproducts or priceless resources, such as carbon dioxide and water, supporting the idea of a circular economy and resource conservation.

### 2.3. Global Research Trends

**Figure 2:**
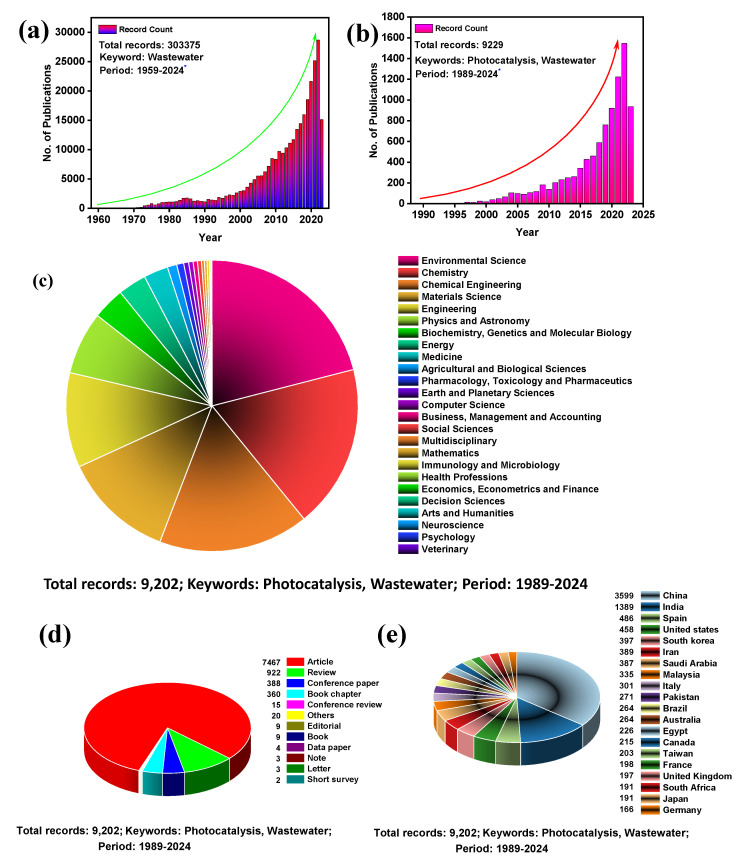
Number of publications for the entire research on (**a**) Wastewater (the topic keywords were set to “Wastewater”) and (**b**) Wastewater and Photocatalysis (the topic keywords were set to “Wastewater, Photocatalysis”); The symbol (*) indicates the value is continuing. (**c**) list of disciplines that are contributing the publication; (**d**) types of publications; and (**e**) list of the top 20 countries contributing the publications. The data were obtained from the SCOPUS database on 28 June 2023.

Wastewater treatment has always been a concern for researchers and environmentalists. Decades of effort have been devoted to discovering a long-term solution to this issue. From the SCOPUS database, we found that wastewater treatment’s foundation was laid as early as 1959. Since then, this field has gained considerable interest from researchers worldwide. An exponential growth can be seen in the number of publications each year with the keyword “wastewater” (Figure 2a). From 1959 to 28 June 2023, a total of 303,375 publications have been published so far with the keyword “wastewater.” Similarly, an exponential growth trend was observed for another SCOPUS database search with the keyword “photocatalysis + wastewater”, with 9229 publications until 28 June 2023 (Figure 2b). All of these 9229 publications were contributed by 25 different disciplines, among which ‘Environmental Science’ contributed 21%, followed by 18% from ‘Chemistry’, and 17% from ‘Chemical Engineering’ (Figure 2c). Out of these publications, the major contributing parts are articles, reviews, and conference papers (Figure 2d). From this data, it is also observed that most Asian countries are significant contributors to this field of research. Among the top 20 countries, only China and India contribute 50% of publications (Figure 2e). Overall, it shows that photocatalytic wastewater treatment is a trending topic in the current era. Herein, we focus on revealing the potential of wastewater treatment by the photocatalytic method using g-C_3_N_4_/TNP nanocomposites, so that it can open new insights for future research aiming towards a sustainable environment.

## 3. g-C_3_N_4_/TNP Nanocomposite as Photocatalysts

### 3.1. Overview of g-C_3_N_4_

**Figure 3:**
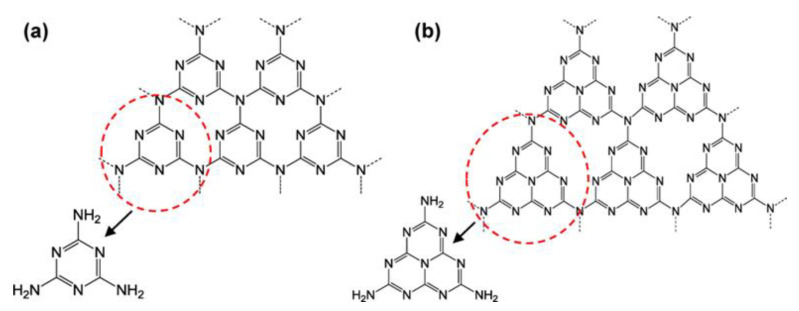
(**a**) Triazine and (**b**) tri-s-triazine structures of g-C_3_N_4_, adapted from ref. [40] with copyright permission from the American Chemical Society.

In 2009, Wang et al. utilized g-C_3_N_4_ for photocatalytic water splitting to produce hydrogen [38]. Since 2009, this material has received considerable attention in photocatalytic applications due to its favorable optical band gap, non-toxicity, affordability, and excellent chemical stability. Moreover, it has been extensively used in the wastewater treatment industry. g-C_3_N_4_ is a 2D layered material composed of nitrogen-doped graphitic layers stacked on top of each other [39]. The two-dimensional (2D) layered structure of g-C_3_N_4_ is similar to that of graphene. Through sp^2^ hybridization, the carbon and nitrogen atoms display a π-conjugated electronic structure and stack on top of one another to create a three-dimensional (3D) crystal structure. The basic structural unit of g-C_3_N_4_ exists in two forms: s-triazine (C_3_N_3_) and tri-s-triazine (C_6_N_7_) (Figure 3). The second one is relatively stable, as shown by density functional theory (DFT) calculations and is thus used as the theoretical model in most studies [40]. The main influencing factor for photocatalytic activity is the energy band architecture. With the conduction band (CB) and valance band (VB) positioned at about 1.1 eV and 1.6 eV, respectively, g-C_3_N_4_ has a band gap of approximately 2.7 eV. g-C_3_N_4_ can be used in visible light because of its maximum absorption wavelength of 460 nm [41]. g-C_3_N_4_ composite materials have higher degradation efficiencies for some organic dyes than TiO_2_ and other semiconductors due to their distinct bandgap structure [42]. Recently, Liu et al. found that the π–π interaction resulted in π* electron transition in carbon–nitrogen homojunctions, which degraded uranium(VI) up to 99.6% in simulated solar radiation [43]. The presence of nitrogen in the g-C_3_N_4_ structure enhances the separation and transfer of photo-induced electron–hole pairs, resulting in an increase in photocatalytic activity [44].

Additionally, it can be synthesized by various methods such as thermal condensation [45,46,47,48], solvothermal [49,50], and hydrothermal [51,52,53] methods. Other synthesis methods, along with important properties and photocatalytic applications, are listed in Table 1. g-C_3_N_4_ has been used in various photocatalytic applications, such as water splitting [48,54,55], CO_2_ reduction [56], and organic pollutant degradation [51,57,58,59]. There are still many issues that need to be fixed in order to enhance its photocatalytic activities. Pure g-C_3_N_4_ is less effective as a solar photocatalyst due to its large band gap and weak visible light absorption. The visible light absorption properties of g-C_3_N_4_ can be improved using various techniques such as elemental doping, hybridization with other substances [60], and/or surface modification [61]. These techniques can increase its photocatalytic performance all around and widen the range of light absorption. The photocatalytic efficiency of g-C_3_N_4_ is limited by the quick recombination of photogenerated electron–hole pairs, which decreases the total number of active charge carriers available for catalytic reactions [62]. The separation of charge carriers can be improved by introducing defects or heterostructures [63] and increasing the surface area. These techniques can reduce charge carrier recombination and increase photocatalytic activity. g-C_3_N_4_ shows poor catalytic performance for some advanced applications such as water splitting, CO_2_ reduction, and organic pollutant degradation. The catalytic activity and selectivity of g-C_3_N_4_ can be improved through rational design and engineering by changing its composition, doping it, or adding co-catalysts [64,65]. Additionally, g-C_3_N_4_ photocatalytic performance can be increased by investigating synergetic interactions with other materials.

### 3.2. Overview of TNP Photocatalysts

**Figure 4:**
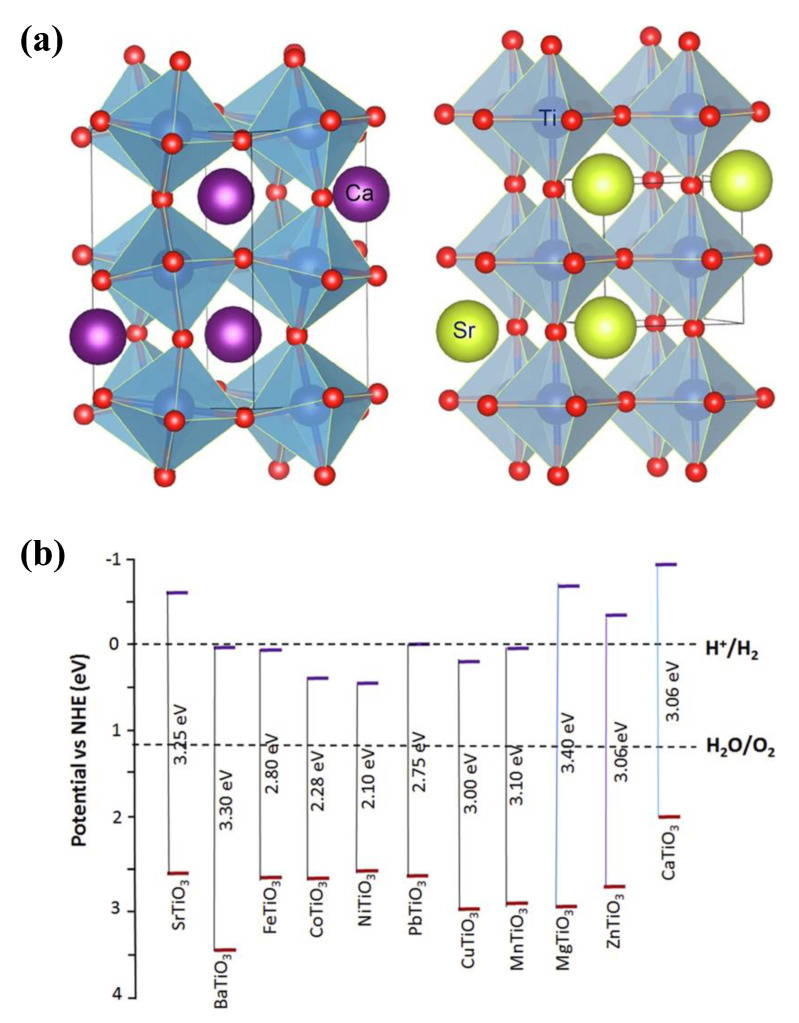
(**a**) Crystal structure of orthorhombic CaTiO_3_ (left) and cubic SrTiO_3_ (right), depicted with VESTA^®^ software (version 3); adapted from ref. [83] with copyright permission from Elsevier. (**b**) Titanate-based perovskites and their bandgaps and band edges with respect to the redox potential of water splitting; adapted from ref. [84] with copyright permission from Elsevier.

The broader family of perovskite compounds includes a unique class of materials known as titanate perovskites (TNPs). Their chemical formula is ATiO_3_, where A stands for a monovalent or divalent cation in the compound [73]. The perovskite crystal structure of TNPs is characterized by a 3D network of corner-sharing octahedrals (Figure 4a). Due to their distinctive structural and electronic properties, perovskite-type titanates have especially drawn significant interest in photocatalysis [74]. These materials are strong contenders for a variety of photocatalytic applications due to their favorable optical absorption characteristics, effective charge carrier separation, and good stability. TNPs have distinct crystal structures and electronic characteristics [75], which easily separate the photogenerated electron–hole pairs, improving photocatalytic performance. TNPs’ photocatalytic activity is controlled by several mechanisms. When light is absorbed, the TNP lattice, electron–hole pairs are produced. The distinct crystal structure and electronic characteristics allow for the spatial separation of the photogenerated electron and hole, delaying the recombination process [27]. The photogenerated charge carriers take part in surface redox reactions that result in the desired photocatalytic transformations, such as water splitting, pollutant degradation, or organic synthesis [76]. Reactive oxygen species (ROS) are a crucial component of photocatalytic activity and are produced when certain TNPs activate molecular oxygen [77]. TNPs have shown promising results in a variety of photocatalytic applications (Table 2). These substances have excellent photocatalytic activity to breakdown organic pollutants, including dyes, pesticides, and emerging contaminants, offering a potential remedy for environmental remediation [78,79,80,81]. Under light irradiation, TNPs can convert carbon dioxide (CO_2_) into chemicals or fuels with a higher value, reducing CO_2_ levels and greenhouse gas emissions [82].

TNPs have great potential for photocatalysis, but several issues need to be resolved. For some TNPs, long-term stability under photocatalytic conditions is still a problem [85]. More efforts are required to increase their resistance to photo-corrosion and degradation. The limited ability to use visible light, which makes up a large portion of solar irradiation, is due to the band gaps of these materials frequently falling in the UV or near-UV range (less than 400 nm wavelength) (Figure 4b) [86,87]. High photocatalytic activity depends on efficient charge carrier separation and the reduction of recombination losses. However, overall efficiency may be limited by charge carrier trapping and recombination procedures. It still needs to be fully comprehended the precise reaction mechanisms and active sites necessary for photocatalytic processes on TNP surfaces. For large-scale industrial applications, the scalability and cost-effectiveness of TNP-based photocatalysts must be addressed. For practical implementation, it is essential to develop scalable synthesis techniques, investigate cheap and abundant precursors, and optimize the catalyst design for resource efficiency. To fully take advantage of the multifunctionality and synergistic effects of TNP-based photocatalysis, it is necessary to understand the complex interactions between the various components.

**Table 2:** Overview of different TNP synthesis methods, important properties, and photocatalytic applications.

TNP Type	Synthesis Method	Morphology	Bandgap (eV)	Application	Ref.
ZnTiO_3_, CdTiO_3_, PbTiO_3_	Solid state; solvo-combustion	Irregular	3.7 4.0 2.75	H_2_ production	[88]
BaTiO_3_, CaTiO_3_, SrTiO_3_	Solid state	Elongated cylinders; spherical	2.89 2.92 2.85	Methyl orange (MO) degradation	[26]
Na/Fe co-doped BaTiO_3_	Solid state	Spherical	2.3	RhB, malchite green (MG) degradation	[89]
SrTiO_3_	Sol–gel	Tubular	3.18	MO degradation	[90]
Ag doped ZnTiO_3_	Sol–gel	Hexagonal	3.54–3.50	MB degradation, antibacterial	[86]
ZnTiO_3_	Sol–gel	Rod	3.54–3.75	Amoxicillin (AMX), TC, MO, MB degradation	[78]
ZnTiO_3_	Sol–gel	Spherical	3.2	MO degradation	[91]
La_2_Ti_2_O_7_	Sol–gel	Large particles	NA	Azophloxine degradation	[79]
Pt/CaTiO_3_	Sol–gel	Cluster	2.8	Photoconversion of nitrobenzene (NTB) to aniline	[80]
SrTiO_3_	Hydrothermal	Nanocubes	3.19	MB, Tartrazine (TZ) degradation	[81]
MTiO_3_ (M = Sr, Ba, Ca)	Hydrothermal	Spherical	3.0–3.2	H_2_ production, MB degradation	[76]
Bi_4_Ti_3_O_12_	Hydrothermal	Spherical	2.79	MO degradation	[25]
Au@PbTiO_3_	Hydrothermal	Nanoplates	3.05	RhB degradation	[92]
PbTiO_3_/CdS	Hydrothermal	Rectangular nanoplates	2.85 (PbTiO_3_), 2.35 (CdS)	H_2_ production	[93]
PbTiO_3_	Hydrothermal	Nanoplates	3.08	H_2_ production, RhB, MB, MO degradation	[27]
Ni@PbTiO_3_	Hydrothermal	Nanoplates	3.07 (PbTiO_3_), 3.25 (NiO)	RhB degradation	[87]
PbTiO_3_	Hydrothermal	Nanoplates	NA	H_2_ production	[94]
Ag doped PbTiO_3_	Hydrothermal	Irregular pores or foramen	3.76–3.38	MB degradation	[95]
NaTaO_3_, SrTiO_3_	CVD	Orthorhombic, cauliflower	3.12–4.01	H_2_ production	[96]
CaTiO_3_-TiO_2_	CVD	Spherical	3.0	H_2_ production	[97]
MgTi_2_O_5_	CVD	Spherical	3.4	PEC water splitting	[98]
LaPO_4_/CdS	Self-assembly	Root nodule	NA	CO_2_ reduction	[82]
Zn/Cr-LDH-Pb_2_Nb_3_O_10_	Self-assembly	Nanosheet	NA	O_2_ production	[99]
Ba_x_Sr_1−x_TiO_3_	Molten salt	Cubic	3.24	RhB degradation	[100]

In recent years, g-C_3_N_4_/TNP composite photocatalysts have attracted significant attention due to their high photocatalytic activity and stability. g-C_3_N_4_/TNP is a type of heterostructure photocatalyst that combines the properties of both g-C_3_N_4_ and TNP-based perovskite materials. The combination of g-C_3_N_4_ and TNPs can form a p–n junction, which can enhance charge separation. As a result, its photocatalytic activity and stability increase as compared to traditional photocatalysts.

### 3.3. Synthesis Routes for g-C_3_N_4_/TNP Nanocomposites

The various components of g-C_3_N_4_/TNP nanocomposites are incorporated to enhance the photocatalytic activity and take advantage of synergetic effects. A few of the synthesis methods are discussed below.

#### 3.3.1. Hydrothermal Method

**Figure 5:**
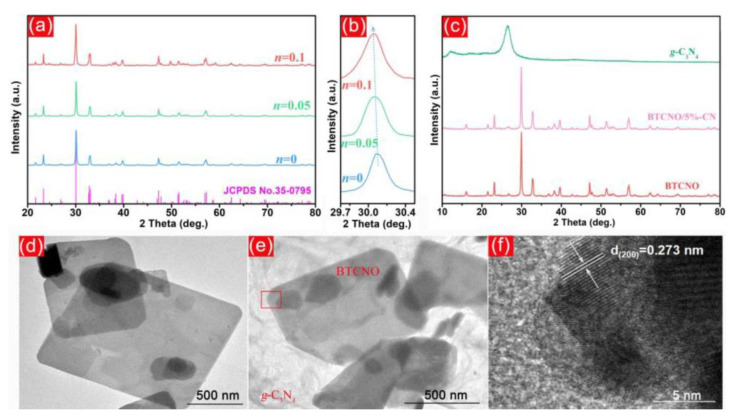
XRD patterns (**a**) and the amplified (1 1 5) peaks (**b**) of BTO and BTCNO with *n* = 0.05 and 0.1 together with those of g-C_3_N_4_, BTCNO, and BTCNO/5%-CN heterojunctions; (**c**) TEM images, (**d**) of BTCNO and (**e**) BTCNO/5%-CN; and (**f**) HRTEM image of BTCNO/5%-CN. Adapted from ref. [31] with copyright permission from Elsevier.

Hydrothermal synthesis methods have received significant attention in photocatalysis because they can create materials with enhanced properties. Typically, this method applies high pressure and high temperature in an aqueous environment, enabling the controlled growth and production of desired crystalline structures [87]. Compared to traditional synthesis techniques, hydrothermal synthesis has several benefits. It gives the reaction conditions a fine level of control, enabling the adjustment of variables including temperature, pressure, and reaction time. These variables are critical in influencing the shape, crystallinity, and surface area of the synthesized materials, all of which directly impact how well they work as photocatalysts [101]. G-C_3_N_4_/TNP photocatalysts can be tailored to have better efficiency, selectivity, and stability by adjusting the hydrothermal conditions [102]. Accordingly, using the hydrothermal method, Bai et al. fabricated a Cr/Nb-modified Bi_4_Ti_3_O_12_/g-C_3_N_4_ [31]. The TEM images showed the successful formation of the composite (Figure 5). Another group of researchers prepared a dual Z-scheme g-C_3_N_4_/Fe_2_TiO_5_/Fe_2_O_3_ ternary nanocomposite using the hydrothermal method. This resulted in the heterogenous distribution of metal nanoparticles on the 2D g-C_3_N_4_ nanosheets [37]. This method helps to retain both the characteristics of g-C_3_N_4_ and TNP, as found from the XRD and TEM results.

#### 3.3.2. Solid-State/Heat Treatment Method

**Figure 6:**
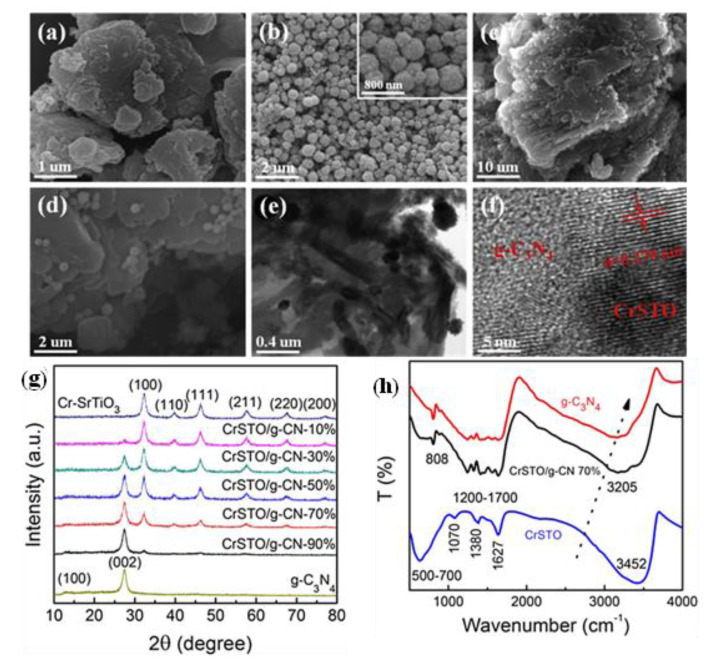
SEM images of (**a**) pure g-C_3_N_4_ nano-sheets, (**b**) pure Cr-doped SrTiO_3_ spheres, and (**c**,**d**) CrSTO/g-CN-70% hybrid nanocomposites. TEM (**e**) and high-resolution TEM (**f**) images of CrSTO/g-CN-70% hybrid nanocomposites. The inset in (**b**) shows a Cr-doped SrTiO_3_ sphere at increased magnification. (**g**) XRD patterns of pure g-C_3_N_4_ nano-sheets, pure Cr-doped SrTiO_3_ spheres, and CrSTO/g-CN hybrid nanocomposites with different mass ratios of 10%, 30%, 50%, 70%, and 90%, respectively. (**h**) FT-IR spectra of pure g-C_3_N_4_ nano-sheets, Cr-doped SrTiO_3_ spheres, and CrSTO/g-CN-70% hybrid nanocomposites. Adapted from ref. [104] with copyright permission from Elsevier.

A versatile and widespread method for producing TNP photocatalysts is the solid-state/heat treatment process. In this method, the constituent materials are mixed in a stochiometric ratio and then mixed using a mortar or ball mill for several hours in an alcoholic medium. The mixture is then subjected to calcination at a high temperature to form the composite. This method is preferable because it neither involves any critical experimental procedures nor complex instrument handling. It is low-cost, and the yield is very high. Yang et al. developed a g-C_3_N_4_/BaTiO_3_ composite using this method of ball milling for 32 h and heating at 300 °C for 1 h [103]. The spherical BaTiO_3_ was uniformly deposited on the 2D g-C_3_N_4_ sheets. The TEM images showed the distinctive interfaces between the two phases, indicating the successful formation of the nanocomposites. Chen et al. prepared a Cr-doped SrTiO_3_/g-C_3_N_4_ hybrid nanocomposite by adding the CrSTO powders and g-C_3_N_4_ nanosheets into a ball mill in an alcoholic medium [104]. After heat treatment, the final product was collected. The XRD pattern of nanocomposites showed the diffraction patterns for both CrSTO and g-C_3_N_4_, which confirmed the successful formation of the composite. The FTIR data also showed all the characteristic peaks of CrSTO and g-C_3_N_4_, which agree well with the XRD data. The SEM images showed the homogenous distribution of CrSTO particles on the g-C_3_N_4_ sheets (Figure 6).

#### 3.3.3. In Situ Method

This method involves the simultaneous formation of g-C_3_N_4_ and TNP components through a single synthetic process. Typically, a precursor mixture containing suitable reagents for both materials is subjected to controlled conditions, enabling the simultaneous growth and integration of the composite structure. Li et al. followed an in-situ precursor method by mixing g-C_3_N_4_ with h′ZnTiO_3_-a′TiO_2_ in methanol to prepare g-C_3_N_4_/h′ZnTiO_3_-a′TiO_2_ [105]. Kumar et al. constructed a dual Z-scheme g-C_3_N_4_/Bi_4_Ti_3_O_12_/Bi_4_O_5_I_2_ following an in situ hydrothermal route for hydrogen evolution and antibiotic degradation [106].

**Figure 7:**
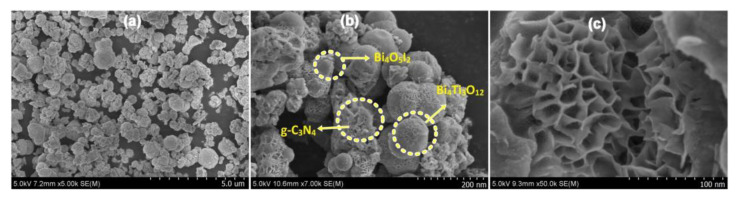
SEM images for CBTB-30 photocatalyst: (**a**) low resolution and (**b**,**c**) high resolution; adapted from ref. [106] with copyright permission from Elsevier.

The morphology (Figure 7) demonstrated an even distribution of spherical and flower-like nanoparticles on the g-C_3_N_4_ sheet. The smooth, spherical particles are Bi_4_Ti_3_O_12_, and the flower-shaped, lamellar surface particles are Bi_4_O_5_I_2_. The formation of the junction is not significantly affected by the morphology of the three moieties. A high-resolution image of Figure 7b makes the close contact of Bi_4_Ti_3_O_12_ and Bi_4_O_5_I_2_ with each other and the rough aggregated stacked sheets of g-C_3_N_4_ even more apparent. In Figure 7c, the junction’s surface can also be seen, with smooth, lamellar, and rough moieties.

#### 3.3.4. Co-Precipitation Method

In this method, aqueous solutions of precursor salts are mixed, and a precipitating agent is added to induce the formation of the resulting composite material. The resulting precipitate is collected, washed, and dried to obtain the composite structure. Recently, a group of researchers produced SrTiO_3_/g-C_3_N_4_/Ag nanocomposites using the co-precipitation method [35].

**Figure 8:**
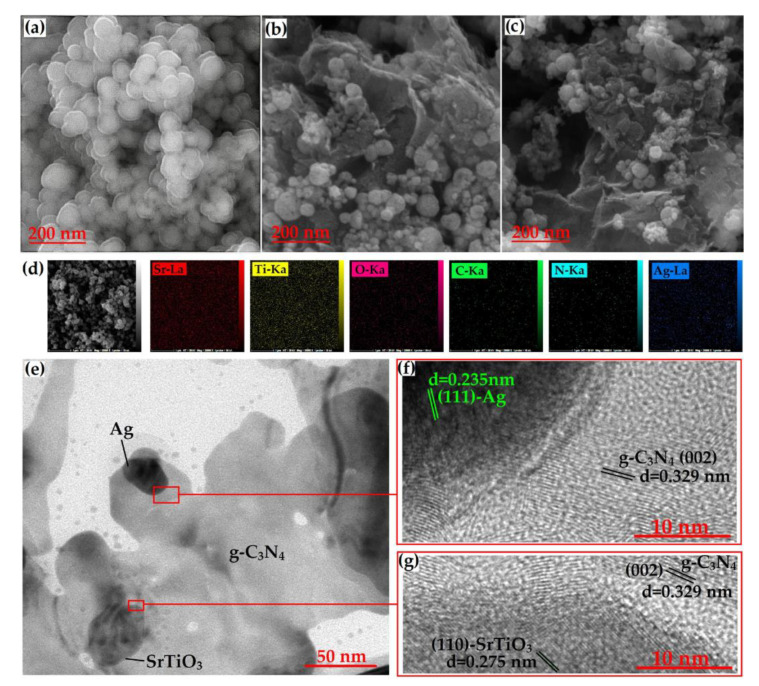
(**a**–**c**) FESEM images of the SrTiO_3_, SrTiO_3_/g-C_3_N_4_, and SrTiO_3_/g-C_3_N_4_/Ag samples, respectively; (**d**) elemental mapping of different elements (Sr, Ti, O, C, N, Ag) present in SrTiO_3_/g-C_3_N_4_/Ag sample; and (**e**–**g**) TEM and HRTEM images of the SrTiO_3_/g-C_3_N_4_/Ag sample. Adapted from ref. [35] with copyright permission from Elsevier.

The average particle size of pure SrTiO_3_, which has been synthesized in spherical form, is about 70 nm. The mean size of SrTiO_3_ particles loaded onto the g-C_3_N_4_ sheets decreased to 59 nm in the FESEM image of the SrTiO_3_/g-C_3_N_4_ binary nanocomposite shown in Figure 8b. By acting as a barrier, g-C_3_N_4_ prevents particle growth, which reduces particle size. The morphology (SEM) of the ternary nanocomposite of SrTiO_3_, g-C_3_N_4_, and Ag is depicted in Figure 8c. Due to Ag nanoparticles, which can serve as a SrTiO_3_ nucleation site, a smaller particle size of 48 nm and a greater amount of agglomeration are seen in the sample. The HR-TEM was carried out at the junctions of the phases (Figure 8f,g) to confirm the synthesis of the anticipated phases. These results demonstrated that the ternary nanocomposite was successfully synthesized.

## 4. Photocatalytic Mechanism

Several complex processes impact the photocatalytic degradation of pollutants using g-C_3_N_4_/TNP nanocomposites, both at the catalyst surface and in the surrounding environment. It is essential to comprehend these mechanisms for the photocatalytic process to run as efficiently and effectively as possible. Traditional photocatalysts have low photocatalytic efficiency because photo-generated electron–hole pairs recombine frequently. The best way to increase electron–hole separation is to build a heterojunction structure [107]. Charge carriers can be easily separated owing to a suitable energy band arrangement between g-C_3_N_4_ and TNPs. g-C_3_N_4_/TNP nanocomposites have higher photocatalytic activity than pure g-C_3_N_4_ or TNPs due to their excellent charge carrier space separation and higher light utilization rate. At this point, various mechanism options are available for designing and fabricating the g-C_3_N_4_/TNP nanocomposites. Some of them are type II heterojunctions, Z-schemes, S-schemes, and p–n junction heterojunctions.

### 4.1. Type II Heterojunction

**Figure 9:**
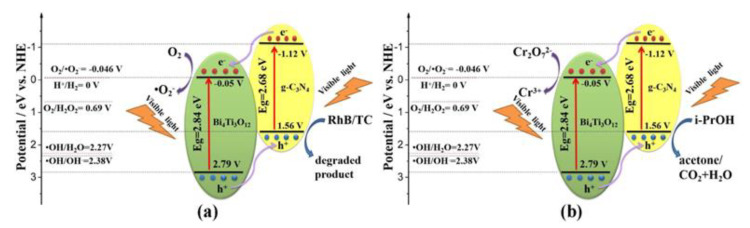
The visible-light photocatalytic mechanisms for (**a**) the degradation of RhB/TC and (**b**) the reduction of Cr(VI) employing CN/BTO composite. Adapted from ref. [109] with copyright permission from Elsevier.

The most common composite heterojunction structure is the type II heterojunction. Due to its benefits, including the lack of a complicated architecture, a variety of simple preparation techniques, and significantly enhanced performance, this heterojunction framework has received much attention. g-C_3_N_4_ and another semiconductor with a low or large band gap comprise type II heterojunctions. Following excitation, photogenerated holes are transferred from the highest VB potential to the lowest VB potential, and photogenerated electrons are transferred from a high CB position to a low CB position. The built-in field also makes it easier for photogenerated charge carriers to separate from one another and relocate [108]. In 2021, Shi et al. synthesized a g-C_3_N_4_/Bi_4_Ti_3_O_12_ composite in which the authors explained the formation of type II heterojunctions (Figure 9) [109]. According to the results of the radical capture tests for holes and free radicals, ●O_2_^−^ and holes were found to be the main reactive species. Once exposed to visible light, both Bi_4_Ti_3_O_12_ and g-C_3_N_4_ could absorb photons, which excited the electrons from the VB to the CB while retaining the holes in the VB. The CB potential of Bi_4_Ti_3_O_12_ (0.05 V) is significantly lower compared to the O_2_/●O_2_^−^ potential (0.046 V vs. NHE). This resulted in the transfer of electrons from the CB of g-C_3_N_4_ to the CB of Bi_4_Ti_3_O_12_ and subsequently reduced O_2_ to ●O_2_^−^, as shown by the energy band structure (Figure 9). The holes from the VB of Bi_4_Ti_3_O_12_ simultaneously moved to the VB of g-C_3_N_4_. Meanwhile, in contrast to the standard reduction potential of ●OH/OH^−^ (2.38 V) or ●OH/H_2_O to form ●OH radicals, the primary degradation mechanism of RhB/TC was due to a direct reaction with the holes. Furthermore, electrons in the CB of Bi_4_Ti_3_O_12_ could reduce Cr_2_O_7_^2−^ to Cr(III) species, while the photoreduced holes of Bi_4_Ti_3_O_12_ headed to g-C_3_N_4_ to oxidize isopropyl alcohol (IPA). The degradation of MO, RhB, and TC, and the reduction of Cr(VI) by CN/BTO nanocomposites showed improved visible light-driven catalytic activities and durability compared to either Bi_4_Ti_3_O_12_ or g-C_3_N_4_ alone, along with reliable stability and durability. Several other researchers, such as Nguyen et al. in 2021 [110], Ashouri et al. in 2023 [111], Chen et al. in 2020 [112], Yang et al. [103], Yan et al. in 2017 [113], and many more, have prepared g-C_3_N_4_/TNP nanocomposites that showed the type II heterojunction mechanism with enhanced photocatalytic activity.

### 4.2. Z-Scheme Heterojunction

**Figure 10:**
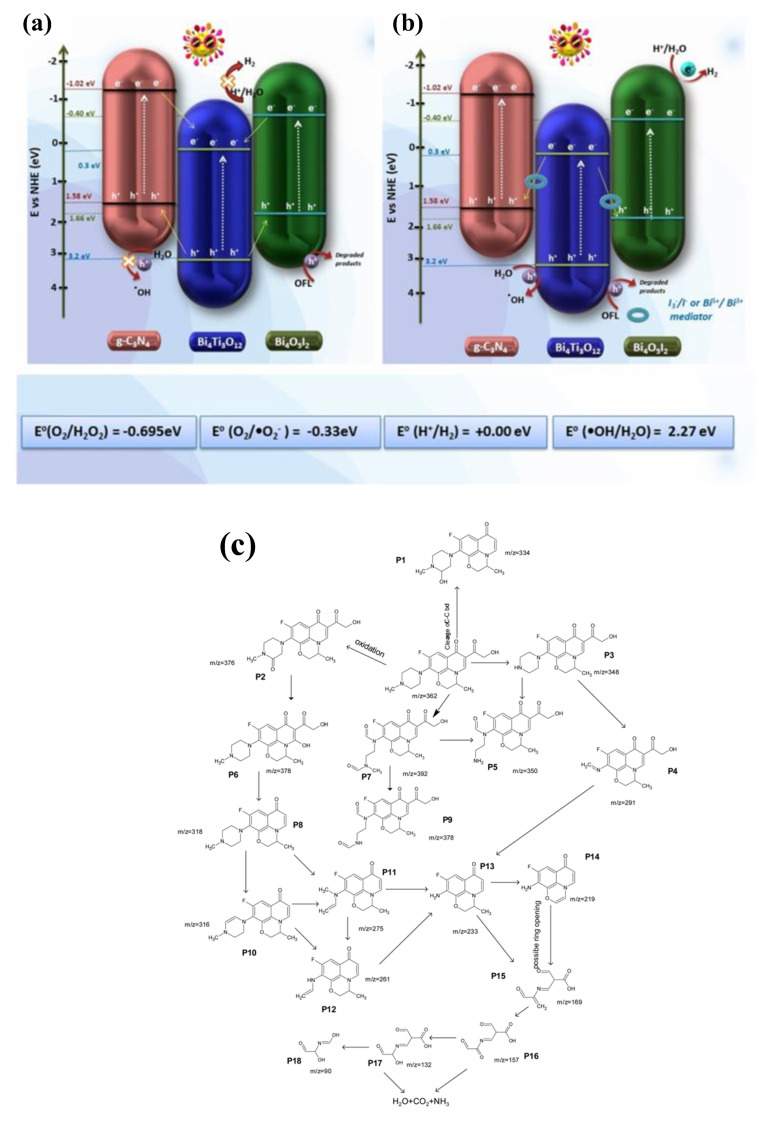
Photocatalytic mechanism for pollutant degradation by (**a**) conventional transfer and (**b**) the dual Z-scheme mechanism; (**c**) possible degradation pathway for SDZ. Adapted from ref. [106] with copyright permission from Elsevier.

In type II heterojunction systems, the photocatalytic redox reaction primarily occurs in the CB at a higher potential (weak reducing ability) and in the VB at a lower potential (weak oxidizing ability). As a result, a weak redox ability (driving force) is formed. On the other hand, in the Z-scheme photocatalytic system, the photocatalytic reaction primarily takes place in CB at a lower potential (strong reducing ability), and in VB at a higher potential (strong oxidizing ability), resulting in better redox ability and photocatalytic activity [107]. Electrons from the semiconductor with a more negative CB can directly combine with VB from the other semiconductor in a Z-scheme heterojunction system [108]. Without using a conductive material as an electron transmission medium, two semiconductors are directly combined to form a heterojunction in a direct Z-scheme heterojunction. The semiconductor material develops a heterojunction structure, so its interface often includes many defects. Following the stacking, the overlap of these defect energy levels could result in creating a quasi-continuous energy level at the interface resembling a metal conductor. The quasi-continuous energy level can transmit photogenerated electrons by effectively separating photogenerated charges using the Z-scheme [107]. Kumar et al. constructed a dual Z-scheme g-C_3_N_4_/Bi_4_O_5_I_2_/Bi_4_Ti_3_O_12_ heterojunction (Figure 10) for antibiotic removal and hydrogen production under visible light [106]. It showed excellent photocatalytic activity of 87.1% against ofloxacin removal. The ternary heterojunction also showed great stability after four cycles, including hydrogen production. Since g-C_3_N_4_ and Bi_4_O_5_I_2_ have close VBs to the CB of Bi_4_Ti_3_O_12_ (BT), the electrons from the CB of BT can transfer quickly to their valance bands. The holes remaining in BT’s highly positive VB can oxidize water into hydroxyl radicals, which break down pollutants. Several other researchers also worked on constructing the Z-scheme heterojunction for g-C_3_N_4_/TNP nanocomposites [31,35,104,105].

### 4.3. S-Scheme Heterojunction

**Figure 11:**
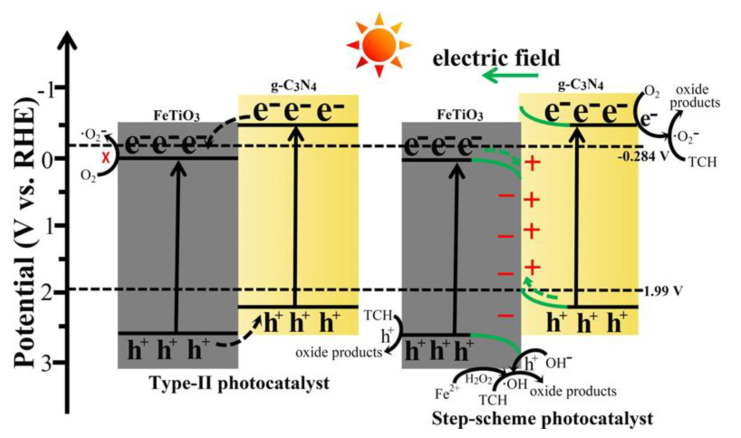
Proposed catalytic mechanism and charge transfer pathways for TCH degradation over FeTiO_3_/g-C_3_N_4_ hybrid systems. Adapted from ref. [116] with copyright permission from Elsevier.

In an S-scheme heterojunction, the prominent photogenerated electrons and holes remain trapped in the CB of the reduction potential and the VB of the oxidation potential, respectively. In contrast, the ineffective photogenerated charge carriers are recombined, introducing a high redox potential [114]. The S-scheme photocatalyst typically consists of two n-type semiconductors, whereas the Z-scheme typically consists of n-type and p-type semiconductors. The combined action of the built-in electric field, Coulomb interaction, and band energy bending functions as the S-scheme photocatalyst [115]. In 2022, Xu et al. created the S-scheme 2D/2D FeTiO_3_/g-C_3_N_4_ hybrid architecture (Figure 11) for the degradation of tetracycline hydrochloride (TCH) [116]. Due to the overlap of the energy band structures of FeTiO_3_ and g-C_3_N_4_, the separation of charge carriers through interfacial transfer in opposing directions was accelerated. A portion of the photogenerated e^−^ of FeTiO_3_ migrated to g-C_3_N_4_ and reacted with the h^+^ in the VB of g-C_3_N_4_, while the remaining photogenerated h^+^ simultaneously migrated to the VB of FeTiO_3_. This is because the CB and VB positions of FeTiO_3_ were lower than those of g-C_3_N_4_ and •O_2_^−^ species produced during the photo-Fenton reaction. The photogenerated h^+^ oxidized OH^−^ to produce •OH species. On the other hand, the Fe^2+^ ions of FeTiO_3_ catalyzed the addition of H_2_O_2_ to produce the •OH species. Over FeTiO_3_/g-C_3_N_4_ hybrid samples, TCH was usefully degraded due to the synergistic interaction of •O_2_^−^ and •OH species. The hybrid composite showed good recyclability up to five cycles, with the best degradation efficiency of 92.6%.

### 4.4. p–n Junction Heterojunction

**Figure 12:**
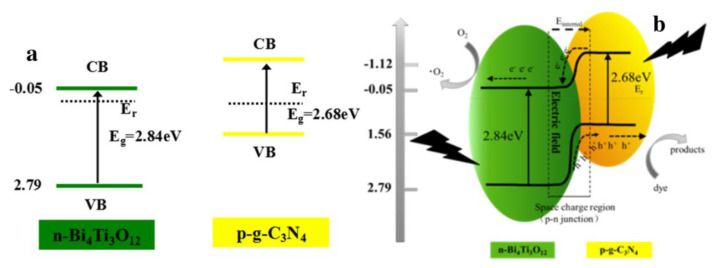
*p–n* heterojunction g-C_3_N_4_/Bi_4_Ti_3_O_12_ prepared through ball milling showing superior photocatalytic activity; energy band structures (**a**) for separate phases and (**b**) after the formation of Bi_4_Ti_3_O_12_ and g-C_3_N_4_ *p–n* heterojunction. Adapted from ref. [117] with copyright permission from Elsevier.

p–n heterojunctions are photocatalysts made from p- and n-type semiconductors. This catalyst can offer an additional electric field to accelerate charge transfer for enhanced photocatalytic activity. The p-type semiconductor material’s holes are transferred to the n-type semiconductor before light irradiation, leaving photogenerated electrons behind. When the fermi-level framework reaches equilibrium, the transfer of electron–hole pairs will stop. Therefore, the p–n heterojunction design can typically increase g-C_3_N_4_′s photocatalytic efficiency [108]. Guo et al. synthesized a g-C_3_N_4_/Bi_4_Ti_3_O_12_ p–n heterojunction (Figure 12) using a simple ball milling technique [117]. The composite decomposed acid orange-7 (AO-7) molecules into CO_2_ and H_2_O as final products. The composite showed excellent recyclability up to four cycles with a minimum degradation of 91.9% from the initial degradation of 95.1%. Energy-rich photons are absorbed by the materials Bi_4_Ti_3_O_12_ and g-C_3_N_4_, which excite the electrons in the VB to the CB and leave holes in the VB. The p-type g-C_3_N_4_′s CB electrons can readily move to the n-type Bi_4_Ti_3_O_12_ because of the band energy structure. The photocatalytic oxidation is started by the electrons in Bi_4_Ti_3_O_12_′s CB, which is n-type. In the VB of n-type Bi_4_Ti_3_O_12_ and p-type g-C_3_N_4_ semiconductors, the orientation of holes undergoes an opposite shift. Thus, charge transfer is assisted by an inner electric field at the junction interfaces between semiconductors with comparable band potentials, which is responsible for the efficient separation of photoexcited electron–hole pairs in p–n junction photocatalysts. Cui et al. [118] followed a similar experiment in 2018 to prepare g-C_3_N_4_/Bi_4_Ti_3_O_12_ with g-C_3_N_4_ nanoparticles (np) and nanosheets (ns). Both types of nanocomposites showed more than 70% degradation for RhB after four cycles, which indicates their good stability, although g-C_3_N_4_(np)/Bi_4_Ti_3_O_12_ showed better results than g-C_3_N_4_(ns)/Bi_4_Ti_3_O_12_.

## 5. Performance of g-C_3_N_4_/TNP Nanocomposites in Wastewater Treatment

Nanocomposites made of g-C_3_N_4_ and TNPs have proven to be incredibly effective at breaking down various organic pollutants found in wastewater (Table 3). When exposed to light, these substances have strong oxidation properties and produce reactive species such as hydroxyl radicals (•OH) and superoxide radicals (•O_2_^−^). These reactive species can break down a variety of organic substances, including pesticides, dyes, pharmaceuticals, and industrial pollutants [116,119,120].

Table 3 shows some g-C_3_N_4_/TNP nanocomposites and their photocatalytic activity including the type of mechanism. For instance, Sohrabian et al. (2023) reported that methylene blue was successfully degraded using a SrTiO_3_/g-C_3_N_4_/Ag composite when exposed to visible light, achieving a high degradation efficiency of over 100% in a short period of time [35]. Kadkhodayan et al. (2023) showed that nZVI-doped Al_2_ZnTiO_9_/g-C_3_N_4_ nanocomposites have increased visible light photocatalytic activity for the degradation of a series of organic pollutants, including toxic heavy metal ions [119]. Similar to this, Kumar et al. (2021) studied the photocatalytic degradation of ofloxacin using a g-C_3_N_4_/Bi_4_Ti_3_O_12_/Bi_4_O_5_I_2_ composite and showed an effective degradation rate of 87.1% within 90 min [106]. In 2020, Yan et al. observed that the CaTiO_3_/g-C_3_N_4_/AgBr ternary heterostructure can photodegrade RhB up to 99.6% [125]. Due to their special structural and electronic characteristics, which make producing and separating photogenerated charge carriers and then activating reactive species for pollutant degradation easier, g-C_3_N_4_/TNP nanocomposites have a high degradation efficiency.

There are several benefits when g-C_3_N_4_/TNP nanocomposites perform against other popular photocatalysts such as TiO_2_ or ZnO. The enhanced visible light absorption of g-C_3_N_4_/TNP nanocomposites makes it possible to use more of the solar spectrum, which is advantageous in indoor and low-light settings. Additionally, they outperform conventional photocatalysts in terms of photocatalytic performance thanks to their unique qualities such as high surface area, tunable bandgaps, and effective charge separation [132]. Additionally, the simplicity of synthesis and modification of g-C_3_N_4_/TNP nanocomposites makes it possible to add co-catalysts and dopants or create composite structures to improve their photocatalytic performance [31,35,104,128,129,130]. Additionally, these materials have good photocatalytic stability, which diminishes the need for constant catalyst replacement and raises the overall cost-effectiveness of the materials.

## 6. Factors Affecting g-C_3_N_4_/TNP Photocatalytic Performance

### 6.1. Charge Carrier Separation, Transfer, and Reactive Species Generation

The g-C_3_N_4_/TNP nanocomposites absorb photons to initiate the photocatalytic reaction. Within the catalyst structure, this excitation causes the generation of electron–hole pairs. For photocatalytic degradation to be successful, these charge carriers must be efficiently separated and transferred [130]. Degradation of the pollutant occurs because of redox reactions involving the separated electrons and holes, and species that have been adsorbed on the catalyst surface. Different reactive species are produced during the photocatalytic process, which are essential for the degradation of pollutants. The hydroxyl radical (•OH), one of the main reactive species, is created when water molecules that have been adsorbed on the catalyst surface react with photogenerated holes (h^+^) [127]. Yang et al. experimented with BaTiO_3_ and g-C_3_N_4_ to determine the main active species responsible for MO dye degradation, in which, after the addition of p-benzoquinone (BQ, 1 mM) (•O_2_^−^ radical scavenger), a strikingly suppressed MO dye degradation is seen. However, when tert-butyl alcohol (TBA, 1 mM, an •OH scavenger) or potassium iodide (KI, 1 mM, scavengers for •OH and h^+^ species) are added, the photocatalytic performance of MO is slightly reduced. These findings indicate that in the g-C_3_N_4_/BaTiO_3_ (10 wt%) composite, the oxidative species •O_2_^−^ is the essential reactive species during the dye degradation process [103].

**Figure 13:**
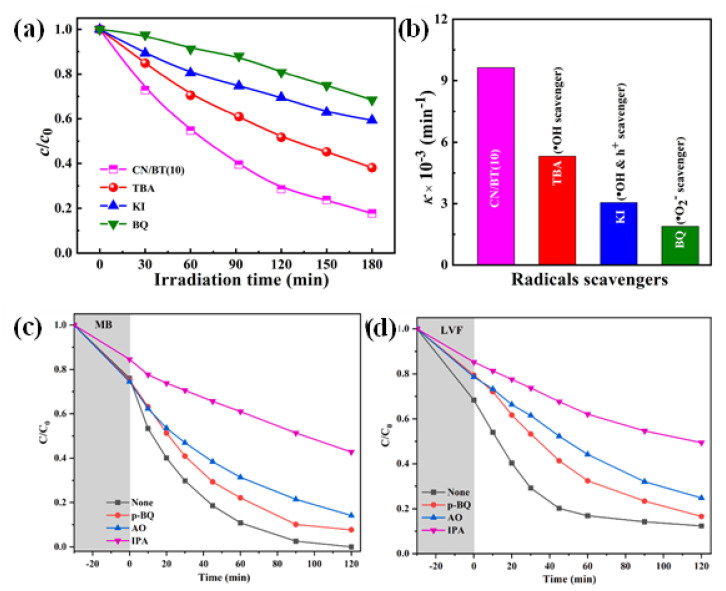
(**a**,**b**) The effect of reactive species during the photocatalytic degradation process (with CN/BT(10) as catalysts); adapted from ref. [103] with copyright permission from Elsevier. Removal rates of (**c**) MB and (**d**) LVF in the presence of scavengers; adapted from ref. [120] with copyright permission from Elsevier.

The highly reactive •OH radical can oxidize organic pollutants, rupturing their chemical bonds. Zhao et al. proposed a radical trapping experiment (Figure 13) using isopropanol (IPA), where •OH acts as a scavenger for the photocatalytic system. As a result, the removal rates of MB and LVF were reduced to about half of the initial removal rate [120]. In addition, the interaction of photogenerated electrons (e^−^) with oxygen species can result in the production of superoxide radicals (•O_2_^−^) and hydrogen peroxide (H_2_O_2_), which further aid in the degradation of pollutants. These reactive species exhibit strong oxidative power, which causes different organic pollutants to deteriorate and become mineralized. 

Depending on the characteristics of the pollutants and the photocatalyst, there are a variety of pathways through which pollutants can be degraded through photocatalysis. One typical pathway involves direct oxidation, in which the adsorbed organic pollutants directly interact with photogenerated reactive species such as •OH or •O_2_^−^ radicals, resulting in the formation of intermediate products and eventually mineralization into harmless by-products such as water and oxygen [133]. Another method is indirect oxidation, in which co-existing substances such as organic pollutants, organic acids, or inorganic ions are oxidized by reactive species produced by the photocatalyst, leading to the degradation of the pollutants. Additionally, some pollutants can go through photocatalytic reduction processes, which involve transferring electrons generated by light to the pollutants, resulting in their reduction to less dangerous forms.

Optimizing the photocatalytic performance of g-C_3_N_4_/TNP nanocomposites requires understanding these photocatalytic mechanisms, including electron–hole separation and transfer, reactive species generation, and pathways for pollutant degradation. Researchers can improve the efficacy and selectivity of pollutant degradation by adjusting the catalyst design, synthesis processes, and reaction conditions, which will help to develop efficient and long-lasting photocatalytic wastewater treatment systems.

### 6.2. Catalyst Loading and Dosage

**Figure 14:**
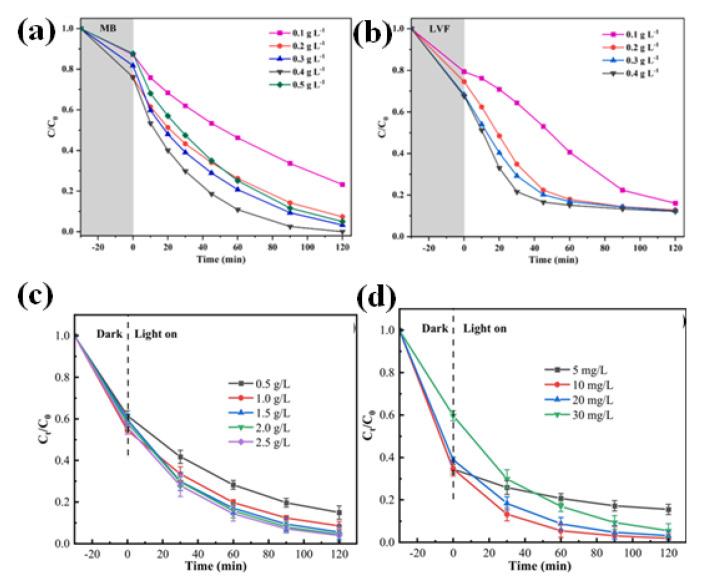
(**a**–**c**) The influence of different dosages of catalyst on photocatalytic activity; Figure a and b adapted from ref. [120], and Figure c adapted from ref. [122] with copyright permission from Elsevier. (**d**) The impact of different concentrations of MG solutions on degradation rate; adapted from ref. [122] with copyright permission from Elsevier.

The performance of the photocatalytic process is significantly influenced by the amount of photocatalyst used or catalyst loading. The right amount of catalyst loading guarantees enough active sites available for pollutant adsorption and subsequent photocatalytic degradation. Inadequate loading may restrict photocatalytic activity, whereas excessive loading may cause active sites to aggregate or be blocked, lowering overall efficiency [117,120,122,134]. Zhao et al. found that when the catalyst dosage increased up to a certain amount, the removal rate of MB also increased to 100% (Figure 14a). High loading of the catalyst further reduced the removal rate. In addition to that, it was also observed that for some pollutants, such as LVF, the dosage amount does not matter that much (Figure 14b) [120]. Zhu et al. (2022) conducted an experiment on CoFe_2_O_4_/g-C_3_N_4_/Bi_4_Ti_3_O_12_ which supports the above-mentioned facts that when the dosage is increased up to a certain amount, it may interact with more active sites beyond which it will either have no effect on the removal rate or decrease it (Figure 14c,d) [122]. To achieve the best performance, careful catalyst loading optimization is required. To balance the degradation efficiency and the treatment system’s cost-effectiveness, the dosage of the photocatalyst used in the wastewater treatment process should also be optimized.

### 6.3. pH

**Figure 15:**
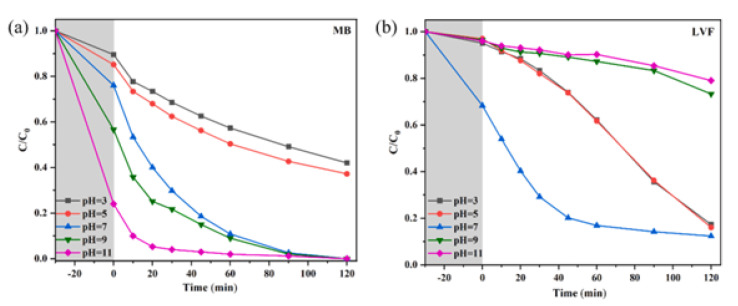
The effect of solution pH on the degradation efficiency of (**a**) MB and (**b**) LVF; adapted from ref. [120] with copyright permission from Elsevier.

pH is a crucial environmental variable that severely affects the photocatalytic degradation process. The ionization state of pollutants and the surface charge of the catalyst are both influenced by pH [106,120,134,135] which impacts how well pollutants adsorb and then degrade. Different pH levels may favor particular reaction pathways and affect the effectiveness of overall degradation. It has been seen that a higher pH may enhance photocatalytic dye degradation such as MB (Figure 15a), but for antibiotics such as LVF, a neutral pH is preferable for their degradation (Figure 15b) [120]. With an increase in pH, the catalyst’s surface electronegativity rises. As a positively charged cationic dye, MB molecules are more readily electrostatically drawn to the composite surface in an alkaline environment. Meanwhile, the literature states that LVF molecules remain cations in solutions with pH less than 6.02, as zwitterions between 6.02 and 8.15, and as anions above 8.15. Since the LVF molecules repel electronegative substances in alkaline conditions, lower removal rates are the result. Kumar et al. studied the effect of pH on the degradation of the antibiotics ofloxacin (OFL) and sulfadiazine (SDZ). It was found that the degradation of OFL is poor in alkaline environments and is best at pH 5. The photocatalyst and OFL have strong electrostatic interactions at pH 5, where the catalyst surface is negatively charged and both OFL and SDZ are positively charged. This results in better adsorption and, as a result, faster degradation. At higher pH, the heterojunction has a negative charge, which limits the adsorption of OH^−^ ions due to repulsion and reduces the production of •OH radicals. Additionally, in a basic medium, airborne CO_2_ is converted into HCO_3_^−^ ions, quenching •OH radicals and forming less CO_3_^−^• radicals [106]. As a result of this discussion, it’s clear that optimizing the pH of the wastewater can improve photocatalytic performance.

### 6.4. Co-Catalysts and Dopants

**Figure 16:**
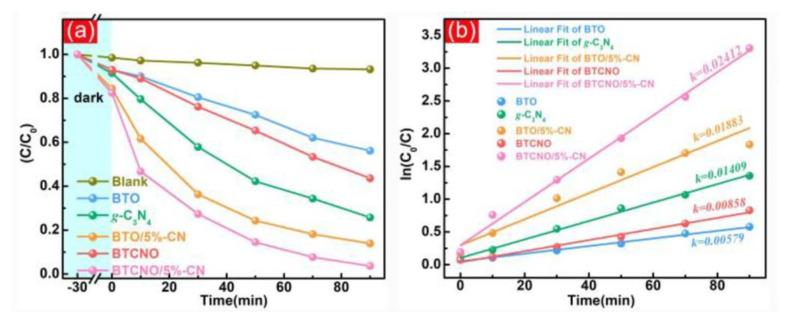
(**a**) Photocatalytic RhB degradation and (**b**) fitted kinetics constants; adapted from ref. [31] with copyright permission from Elsevier.

The performance of g-C_3_N_4_/TNP nanocomposites as photocatalysts can be significantly improved by adding co-catalysts and dopants. Noble metals (such as Pt and Au) [128,130] or metal oxides (such as TiO_2_) [105], which are co-catalysts, can improve charge carrier separation and transfer, increasing the overall photocatalytic efficiency. The performance of catalysts can be altered by the addition of dopants, which changes catalyst crystal structures and electronic characteristics. This may result in increased photocatalytic activity, an extended spectral response, and improved light absorption. Bai et al. introduced Cr and Nb doping into Bi_4_Ti_3_O_12_/g-C_3_N_4_ nanocomposites, resulting in exceptional photocatalytic RhB removal along with hydrogen production (Figure 16) [31]. For the nanocomposite’s photocatalytic performance to be maximized, choosing the right co-catalysts and dopants and optimizing their concentrations are essential.

The photocatalytic performance of g-C_3_N_4_/TNP nanocomposites can be significantly improved by considering and optimizing these variables, including catalyst loading and dosage, pH, and the use of co-catalysts and dopants. Researchers can tailor the synthesis and application of these nanocomposites for particular wastewater treatment scenarios through careful optimization and understanding of these factors, resulting in more efficient and effective pollutant removal from water systems.

## 7. Potential Impact and Significance

In photocatalytic wastewater treatment, the use of g-C_3_N_4_/TNP nanocomposites has enormous potential for reducing water pollution and achieving sustainable water management. The unique properties and performance of these nanocomposites have several significant impacts and contributions to the field:

### 7.1. Environmental Impact

Numerous pollutants can be significantly reduced in water bodies by photocatalytic wastewater treatment using nanocomposites of g-C_3_N_4_/TNP. Pharmaceuticals, emerging contaminants, and organic dyes can be effectively broken down and mineralized to prevent their release into the environment, minimizing the ecological impact on aquatic ecosystems. There are several instances where g-C_3_N_4_-based nanocomposites showed selectivity and specificity for targeted pollutants, enabling efficient removal while minimizing the degradation of irrelevant compounds [136]. This selectivity will be beneficial for the removal of specific contaminants from complex wastewater systems with multiple pollutants without causing any damage to overall water quality. The g-C_3_N_4_/TNP nanocomposites show good degradation ability towards various pollutants, including various dyes, antibiotics, and toxic heavy metals. This reflects their outstanding adaptability to different pollutants. These nanocomposites provide a green and sustainable approach to water treatment by minimizing chemicals and energy-intensive techniques and using solar energy [37] as the catalyst for the photocatalytic process. Utilizing renewable resources and the nanocomposites’ potential for recycling and reusing further helps minimize wastewater treatment procedures’ environmental impact.

### 7.2. Water Resource Conservation

Utilizing g-C_3_N_4_/TNP nanocomposites for wastewater treatment helps preserve and safeguard water resources. These nanocomposites help to maintain water quality by removing pollutants and contaminants from wastewater, ensuring the availability of clean water for various uses such as drinking water supply, agricultural irrigation, and industrial processes. Using nanocomposites offers a sustainable solution for maintaining water resources and lowering reliance on freshwater sources, considering growing concerns about water scarcity around the world.

### 7.3. Public Health and Safety

Public health and safety are improved by removing pollutants and newly emerging contaminants from wastewater using g-C_3_N_4_/TNP nanocomposites. Wastewater contaminants, such as organic dyes, drug remnants, and micropollutants, can harm the environment and human health. The nanocomposites assist in lowering the potential risks related to exposure to harmful substances by efficiently destroying and removing these contaminants. Advanced wastewater treatment ensures the well-being of communities and promotes public health by providing clean and safe water resources.

### 7.4. Economic Opportunity

Economic opportunities are created in numerous sectors due to the development and application of g-C_3_N_4_/TNP nanocomposites in photocatalytic wastewater treatment. More opportunities exist for innovation, research, and development because of the rising demand for advanced water treatment technologies. Systems for photocatalytic treatment must be built, deployed, and operated by skilled workers, which creates employment opportunities. Decentralized and affordable water treatment solutions can also be aided by locally accessible resources, such as an abundance of sunlight, especially in areas without easy access to conventional infrastructure.

### 7.5. Technological Advancement

Technology advancements in the field are driven by the study and use of g-C_3_N_4_/TNP nanocomposites in photocatalytic wastewater treatment. Improvements in performance and efficiency are being made thanks to the development of new synthesis techniques, composite designs, and insights into the mechanisms of photocatalysis. Additionally, combining these nanocomposites with other cutting-edge treatment methods and investigating hybrid systems encourages interdisciplinary cooperation and knowledge sharing. The development of these technologies aids in the creation of environmentally friendly methods for treating water, which benefits not only wastewater treatment but also related industries such as renewable energy and materials science.

To sum up, photocatalytic wastewater treatment using nanocomposites made of TNP and g-C_3_N_4_ has much potential for addressing water pollution issues, achieving sustainable water management, and protecting water resources and public health.

## 8. Challenges and Future Prospects

g-C_3_N_4_/TNP nanocomposites have significantly advanced the field of photocatalytic wastewater treatment. However, various challenges restrict their practical applications. First, the reduction of stability under prolonged exposure to photocatalytic reactions due to catalyst deactivation, aggregation, or leaching of active species may take place, which can diminish wastewater photocatalytic performance. Therefore, the enhancement of stability and recyclability of composite materials needs to be improved through surface modification, encapsulation activity, and the addition of intense cocatalyst materials that can prevent catalyst degradation and enhance the recovery process of catalysts. Second, the large charge separation and compressed light absorption ability of these materials can minimize the photocatalytic activity for wastewater treatment. These issues can be improved by the incorporation of novel nanomaterials, including metal nanoparticles, metal oxides, or carbon-based materials; the doping of heteroatoms; and the addition of intense semiconductor materials. Third, calling up photocatalytic applications from the laboratory scale to the industrial scale brings another challenge. Despite extensive research on the performance of g-C_3_N_4_/TNP nanocomposites in small-scale systems, there needs to be more evidence of their use in large-scale wastewater treatment procedures. Therefore, essential factors such as feasibility, scalability, synthesis processes, and reactor designs need to be carefully taken into account. Furthermore, the overall effectiveness and applicability of photocatalytic wastewater treatment systems can be increased through the incorporation of g-C_3_N_4_/TNP nanocomposites with other processes such as cutting-edge technologies, membrane filtration, adsorption, or electrochemical processes that can intensify the removal of pollutants. Moreover, the combination of these nanocomposite materials can also be implemented in other fields, including renewable energy sources (solar panels or other light-harvesting devices) and environmental applications. Thus, various aspects need further detailed study, including intense selectivity and photocatalytic activity, optimization of operating parameters of photocatalytic materials for their wide practical applications, and modified infrastructure of wastewater treatment systems. There is an essential need to improve the degradation of certain types of pollutants, such as emerging contaminants and intractable organic compounds, that can improve the photocatalytic performance of wastewater treatment. Thus, the above challenges are essentially needed for future detailed studies, including stability, scale-up, selectivity enhancement, and improvement activities for environmental/economic fields. The advancement of water treatment technologies will enable the effective and sustainable removal of pollutants through the creation of high-performance, stable, and cost-effective nanocomposites with specialized properties. Thus, g-C_3_N_4_/TNP nanocomposites have the great potential to revolutionize wastewater treatment, which aids in preserving water resources.

## 9. Conclusions

In photocatalytic wastewater treatment, the combination of g-C_3_N_4_/TNP nanocomposites has emerged as a promising approach for removing various pollutants. In this review, we have emphasized the significant advancements in the synthesis as well as the use of g-C_3_N_4_/TNP nanocomposites, accompanied by their most recent developments and valuable applications in wastewater treatment. The synergetic effects of these nanocomposites have improved photocatalytic performance and extended spectral responses that allow for the effective degradation of various pollutants. The use of these composite materials for water purification, hybrid treatment systems, and industrial and municipal wastewater treatment holds promise for addressing water pollution issues. These composite materials are suitable for other applications, such as the treatment of organic dyes, pharmaceuticals, and emerging contaminants, owing to their improved charge separation, expanded light absorption range, and selective pollutant degradation. However, several difficulties still exist in photocatalytic wastewater treatment based on g-C_3_N_4_/TNP nanocomposites due to their poor stability, recyclability, and reduced functionality. Therefore, these challenges need further comprehensive studies in the future. The combination of g-C_3_N_4_/TNP nanocomposites can contribute to sustainable wastewater management practices, ensuring the availability of clean water resources in the future. Furthermore, integrating these nanocomposites with other advanced technologies, considering environmental and economic factors, and collaborating between researchers and policymakers are critical for successfully implementing photocatalytic wastewater treatment systems on a larger scale. Thus, g-C_3_N_4_/TNP nanocomposites can be considered a potential candidate for effective and selective photocatalytic wastewater treatment.

## Figures and Tables

**Table 1:** Overview of g-C_3_N_4_ synthesis methods, important properties, and photocatalytic applications.

Precursor	Synthesis Method	Morphology	Bandgap (eV)	Application	Ref.
**Urea**	Thermal polymerization	2D lamellar	NA	Carbamazepine degradation	[46]
Melamine, NH_4_Cl	Thermal polymerization	2D nanosheets	2.71	2,4-Dichlorophenol degradation	[45]
Melamine	Thermal polymerization	NA	2.75–2.62	NA	[47]
Melamine	Thermal polymerization	2D nanosheets	2.6–2.49	H_2_ production	[48]
Urea	Hydrothermal	Nanosheets	2.58	Tetracycline (TC) degradation	[51]
Dicyandiamide	Hydrothermal calcination	Laminated hexagonal prisms	2.62	Rhodamine B (RhB) degradation, H_2_ production	[19]
Urea	Thermal polymerization	Sheets	2.8	CO_2_ reduction, H_2_ production	[56]
Thiourea	Thermal polymerization/Solvothermal	Sheets	2.76	H_2_ production	[49]
Urea, thiourea	Calcination	Nanoflakes	2.78–2.89	RhB degradation	[57]
2,4,6-Trichloro-1,3,5-triazine, dicyandiamide, acetonitrile	Solvothermal	Spherical	2.19	Tetracycline hydrochloride degradation	[50]
Melamine	Hydrothermal	Nanotubes	2.70	NO removal	[52]
Melamine, ammonium thiosulfate	Hydrothermal	Lamellar	2.64	H_2_ production	[53]
Melamine	Thermal polymerization/Hydrothermal	NA	2.3–2.7	Methylene blue (MB) degradation	[58]
Melamine, calcium cyanamide	Template assisted	Stacked lamellar	2.66	H_2_ production	[54]
Urea, glucose, P123	Template assisted	Curled sheets	NA	Energy storage	[66]
Dicyandiamide, NaCl	Template assisted	Honeycomb	2.6	H_2_ production	[55]
Melamine, magadiite	Template assisted	Layered	2.8	RhB degradation	[67]
Melamine, artificial graphite powders	Microwave	Layered	NA	RhB, methyl orange (MO) degradation	[59]
Melamine, carbon fiber	Microwave	Nanosheets	2.88	Field emission	[68]
Urea	Microwave	Nanosheets	NA	White LED	[69]
Cyanuric chloride, sodium azide	Microwave	Spherical	2.41	NA	[70]
Thiourea	Microwave	Nanoplates	2.7–2.61	Nitrogen photofixation, RhB degradation	[71]
Citric acid, thiourea	Microwave	NA	NA	Fluorescence probe	[72]

**Table 3:** Synthesis method of g-C_3_N_4_/TNPs nanocomposites and their photocatalytic activities for wastewater treatment.

Photocatalysts	Synthesis Method	Heterojunction Type	Light Source	Photocatalytic Activity/Rate Constant, k (min^−1^)	Main Active Species	Ref.
Cr/Nb-modified Bi_4_Ti_3_O_12_/g-C_3_N_4_	Hydrothermal	Z-scheme	300 W Xe lamp	98.7% of RhB degradation	●OH, ●O_2_^−^	[31]
nZVI-doped Al_2_ZnTiO_9_/g-C_3_N_4_	Hydrothermal	NA	70 W Xe arc lamp	88%, 87%, 80%, 72%, and 90% degradation for methyl orange anion dye, methylene blue cation dye, nitrate, carbon dioxide, and toxic heavy metals, respectively	●O_2_^−^, ●OH	[119]
g-C_3_N_4_/Bi_4_Ti_3_O_12_/Bi_4_O_5_I_2_	In situ hydrothermal	Z-scheme	500 W Xe lamp	87.1% ofloxacin removal	h^+^, ●OH	[106]
g-C_3_N_4_/Bi_4_Ti_3_O_12_	Thermal polymerization	Heterostructure	300 W Xe lamp	96.99% of RhB. 84.20% of TC, 69.64% Cr(iv) reduction	h^+^, ●O_2_^−^	[109]
SrTiO_3_/g-C_3_N_4_/Ag	Co-precipitation	Z-scheme	400 W OSRAM lamp	100% MB degradation	h^+^,●O_2_^−^, ●OH	[35]
g-C_3_N_4_/Fe_2_TiO_5_/Fe_2_O_3_	Hydrothermal	Z-scheme	Sunlight	96.1% MB degradation (k = 0.009)	●O_2_^−^, ●OH	[37]
g*-*C_3_N_4_/BaTiO_3_	Mixing–calcining	Heterostructure	100 mW/cm^2^ xenon lamp equipped	MO degradation	●O_2_^−^	[103]
g-C_3_N_4_/h′ZnTiO_3_-a′TiO_2_	In situ	Z-scheme	350 W Xe arc lamp	99.8% MB degradation	●O_2_^−^, ●OH	[105]
Cr-SrTiO_3_/g-C_3_N_4_	Solid state	Z-scheme	500 W Xe lamp	97% of RhB degradation	h^+^, ●O_2_^−^	[104]
g-C_3_N_4_/SrTiO_3_	Sonication mixing	Z-scheme	2.2 kW Xe lamp, LED flood lamps	MB degradation (k = 0.0220)	●OH, ●O_2_^−^	[121]
CaTiO_3_/g-C_3_N_4_	Solid state	Z-scheme	500W mercury lamp	92.7% MB, 87.7% levofloxacin degradation	●OH	[120]
g-C_3_N_4_/Bi_4_Ti_3_O_12_	Ball milling	p–n junction	500 W Xe lamp	87.2% AO-7 degradation	h^+^, ●O_2_^−^	[117]
CoFe_2_O_4_/g-C_3_N_4_/Bi_4_Ti_3_O_12_	Ultrasonic-assisted heat treatment	Z-scheme	45 W energy-saving lamp	98.05% degradation of MG	h^+^, ●O_2_^−^	[122]
Bi_4_Ti_3_O_12_/g-C_3_N_4_/BiO_5_Br	Thermal polymerization	Z-scheme	65 W energy-saving lamp	89.84% TC degradation	●O_2_^−^	[123]
g-C_3_N_4_/N-doped LaTiO_3_	Solid state	Heterostructure	100W halogen lamp	90% of RhB degradation	●O_2_^−^, ●OH	[124]
CaTiO_3_/g-C_3_N_4_/AgBr	Mixing	Z-scheme	200 W Xe lamp	99.6% of RhB degradation	●O_2_^−^	[125]
g-C_3_N_4_/La_2_Ti_2_O_7_	Wet impregnation	Heterostructure	400 W Xe lamp	MB degradation	h^+^, ●O_2_^−^	[126]
SrTiO_3_/g-C_3_N_4_	Thermal treatment	Heterostructure	Six fluorescent lamps	MB degradation (k = 1.30 × 10^−3^), amiloride (AML) degradation (k = 1.82 × 10^−3^)	●OH	[127]
Pt/g-C_3_N_4_/SrTiO_3_	Low-temperature calcination	Z-scheme	500 W Xe lamp	93% acid red 1 (AR1) dye degradation	●O_2_^−^	[128]
FeTiO_3_/g-C_3_N_4_	self-assembly	S-scheme	300 W Xe lamp	92.6% tetracycline hydrochloride degradation	h^+^, ●O_2_^−^, ●OH	[116]
2D/1D g-C_3_N_4_/CaTiO_3_	Solvothermal	Heterostructure	300 W Xe lamp	99.76% crystal violet (CV) and 95.02% MG degradation	h^+^, ●O_2_^−^	[112]
g-C_3_N_4_/Bi_12_TiO_20_	Annelation	Heterostructure	500 W Xe lamp	96.9% of RhB degradation	h^+^, ●O_2_^−^	[113]
g-C_3_N_4_/Bi_4_Ti_3_O_12_	Mixing–calcining	p–n heterojunction	200 W Xe lamp	85.4% RhB degradation	h^+^, ●O_2_^−^	[118]
SrZnTiO_3_/g-C_3_N_4_	Mixing–calcining	Z-scheme	500 W halogen lamp 400 W high pressure mercury lamp	93.1 and 82.2% removal of indigo carmine (IC) and RhB, respectively	h^+^,●O_2_^−^, ●OH	[129]
PbTiO_3_/g-C_3_N_4_	Mixing–calcining	Heterostructure	300 W UV Xe lamp	RhB degradation (k = 0.1357)	NA	[130]
*g*-C_3_N_4_/BaTiO_3_	Hydrothermal	Heterostructure	75 W–220 V lamp	98.72% MB degradation	●O_2_^−^, ●OH	[110]
NiTiO_3_@g-C_3_N_4_	Ultrasonic-assisted wet-impregnation	Heterostructure	Direct sunlight	95−98% photoreduction of toluene to benzoic acid	●OH	[131]
La_2_Ti_2_O_7_/C_3_N_4+x_H_y_	Hydrothermal	Heterostructure	240 W mercury lamp	Degradation of 99, 95 and 93% for RhB, MB, and MO, respectively	h^+^, ●O_2_^−^	[111]

## Data Availability

Data are contained within the article.
